# Gene expression analysis of heat-shock proteins and redox regulators reveals combinatorial prognostic markers in carcinomas of the gastrointestinal tract

**DOI:** 10.1016/j.redox.2018.11.018

**Published:** 2018-11-29

**Authors:** Sebastian Öther-Gee Pohl, Shazib Pervaiz, Arun Dharmarajan, Mark Agostino

**Affiliations:** a Stem Cell and Cancer Biology Laboratory, Curtin University, Perth, WA, Australia; b School of Pharmacy and Biomedical Sciences, Curtin University, Perth, WA 6102, Australia; c Curtin Health and Innovation Research Institute, Curtin University, Perth, WA 6102, Australia; d Department of Physiology and Medical Science Cluster Cancer Program, Yong Loo Lin School of Medicine, National University of Singapore, Singapore 117593, Singapore; e NUS Graduate School for Integrative Sciences and Engineering, National University of Singapore, Singapore 117456, Singapore; f National University Cancer Institute, National University Health System, Singapore 119074, Singapore; g Curtin Institute for Computation, Curtin University, Perth, WA 6102, Australia

**Keywords:** Heat shock proteins (HSPs), Redox regulators, Gene expression analysis, Gastrointestinal carcinomas

## Abstract

Heat shock proteins (HSPs) are a large family of ubiquitously expressed proteins with diverse functions, including protein assembly and folding/unfolding. These proteins have been associated with the progression of various gastrointestinal tumours. Dysregulation of cellular redox has also been associated with gastrointestinal carcinogenesis, however, a link between HSPs and dysregulation of cellular redox in carcinogenesis remains unclear. In this study, we analysed mRNA co-expression and methylation patterns, as well as performed survival analysis and gene set enrichment analysis, on gastrointestinal cancer data sets (oesophageal, stomach and colorectal carcinomas) to determine whether HSP activity and cellular redox dysregulation are linked. A widespread relationship between HSPs and cellular redox was identified, with specific combinatorial co-expression patterns demonstrated to significantly alter patient survival outcomes. This comprehensive analysis provides the foundation for future studies aimed at deciphering the mechanisms of cooperativity between HSPs and redox regulatory enzymes, which may be a target for future therapeutic intervention for gastrointestinal tumours.

## Graphical abstract

**Figure:**
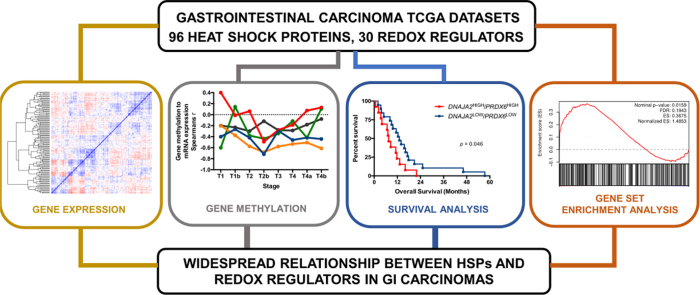

## 1 Introduction

Heat shock proteins (HSPs) are a highly conserved, ubiquitously expressed family of proteins, that act as molecular chaperones to correctly fold a new polypeptide chain, or support the refolding of damaged proteins [1]. HSPs have been implicated in a variety of cellular processes, including cell cycle regulation, organelle trafficking, and signal transduction [2]. Interestingly, experimental evidence indicates that stress-induced activation of HSPs endows cells with a survival advantage by mechanism(s) that, on the one hand, activate pro-survival networks, and on the other, inhibit apoptotic execution [3]. These attributes and the association with pro-inflammatory signalling lend credence to the involvement of HSPs in promoting carcinogenesis and its progression [4], [5]. Along similar lines, the differential expression of various HSP family members and dysregulation of redox homeostasis have been individually implicated in tumorigenesis, particularly involving the gastrointestinal (GI) tract [6], [7]. However, the interplay between HSPs and redox regulation in the setting of GI cancers is less widely understood.

HSPs are divided into five families, including the DNAJ (HSP40) family, the HSPB (small heat shock protein family), the HSP90/HSPC family, chaperonins (HSPD/E and CCT) and the HSP70 superfamily, containing the HSPA (HSP70) and HSPH (HSP110) subfamilies [8]. These families mainly differ in their functions and cellular localisations. The DNAJ/HSP40 family primarily function by stimulating the ATPase activity of HSP70s [9], while the HSP70 family has been implicated in most aspects of protein folding, import, recovery from aggregation and drug resistance in cancer [10]. HSP90 is involved in many cellular processes, including DNA repair [11], and has been implicated in a variety of neoplasia [12]. The HSPB family, in particular HSP27, has been shown to be associated with several human cancers, as it promotes epithelial to mesenchymal transition (EMT) [13], [14], blocks apoptotic signalling [15], and triggers activation of oncogenic transcription factors such as NF-κB [13] and mitogen activated protein kinases (MAPK) [16], independent of their ATP hydrolysis function [17]. Chaperonins, or CCT (chaperonin containing TCP-1) proteins, are ring complexes encompassing a central cavity; this allows protein folding in a shielded environment. They can be divided into two groups: Group 1 (HSPD), that are present in the mitochondria, and Group 2, that are localized to the cytosol [18].

An imbalance in intracellular redox conditions can promote or inhibit tumour growth [19]. This is dependent on a tight balance between the different reactive oxygen species present within the cell [20]. Increased superoxide (O_2_^-^) production, favouring a pro-oxidant milieu, has been demonstrated to act as a pro-survival signal in some cancers [21], [22], [23], while increased peroxides, particularly hydrogen peroxide (H_2_O_2_), can initiate death signalling, leading to apoptosis [24], [25]. There is a close relationship between intracellular redox and HSPs. Protein folding, specifically, disulfide bond formation, is an oxidative process and generates ROS, primarily in the form of H_2_O_2_ [26], [27]. Dysregulation of protein folding can cause oxidative stress and stimulate the unfolded protein response (UPR), which is dependent on HSP proteins [28]. Furthermore, ROS-mediated DNA oxidation and lipid peroxidation can result in the induction of HSP synthesis [29]. For example, a redox-regulated HSP family member, HSP33, functions as a cellular protective agent against oxidative stress in prokaryotes; a coordinated zinc ion of HSP33 is released from the protein following the oxidation of cysteine residues by H_2_O_2_ to activate the protein [30]. Mammalian HSP1 has been reported to exhibit a similar oxidative stress response to prokaryotic HSP33. Two conserved cysteine residues near the DNA-binding domain are crucial for the formation of higher order homomultimers, required for target gene transcription and protection from oxidative stress [31]. These redox-mediated reversible reactions are critical for the activity of HSP33 and HSP1 and demonstrate the importance of cellular redox state on HSP function.

Datasets describing molecular and clinical features of patient tumours have been made publicly available through consortia such as The Cancer Genome Atlas (TCGA), accessible through websites such as cBioPortal [32], [33]. These datasets contain large numbers of patient samples across many cancer types, linking mutation, copy-number alterations, transcriptomic, proteomic and methylomic data with clinical attributes, including survival outcomes, age, gender and therapeutic interventions. These large datasets have provided the foundation for studies that have resulted in the successful identification of biomarkers, development of *in vivo* models and the identification of drug candidates [34], [35], [36]. The generation of a gene signature in response to different drugs using only public databases enabled the repurposing of niclosamide ethanolamine, previously used as an anti-helminthic, as an anti-tumour agent against hepatocellular carcinoma (HCC); this molecule disrupted the interaction between cell division cycle 37 and HSP90, and was effective at reducing tumour burden *in vivo* [34]. Similarly, an *in silico* approach using public datasets identified protein tyrosine kinase PTK7 as a potential oncogenic driver in non-small cell lung cancer (NSCLC); these findings were validated *in vitro* and *in vivo*, where PTK7 was found to regulate MKK7-JNK signalling, indicating its utility as a potential therapeutic target [35].

In this study, we identified a range of previously unreported genes in GI cancer patients – specifically, oesophageal, stomach and colorectal cancers – that resulted in significantly altered survival outcomes when differentially expressed. We have also identified new combinations of HSP and redox genes that, when differentially expressed, predicted overall and disease-free survival in the selected cohorts. These genes may represent clinically relevant targets for use as biomarkers in monitoring disease progression and severity, as well as offering potential interest for the development of new cancer therapeutics.

## 2 Methods

### 2.1 Data collection

Gastrointestinal carcinoma datasets were obtained from the relevant TCGA studies via cBioPortal [33] in November 2017, specifically the oesophageal carcinoma [37], stomach adenocarcinoma [38] and colorectal adenocarcinoma data sets [39]. Based on the families set out in the guidelines for the nomenclature for HSPs, we examined 96 HSP genes [8] and 30 cancer-associated genes involved in redox generation and detoxification [40] in a range of analyses, including co-expression of HSP and redox regulator genes, correlation of gene methylation and expression at different cancer stages, survival, and enrichment of other gene families in susceptible populations. For all analyses, the three cancer types were analysed separately. Analysis was facilitated via Perl scripts developed in-house.

### 2.2 Co-expression and methylation analysis

For co-expression analyses, Spearman's ρ values were calculated using mRNA expression *z-*scores (calculated from RNA Seq V2 RSEM data, compared to the expression distribution of each gene in tumours that are diploid for that gene, as provided by cBioPortal) for all gene combinations, using all patient data. Spearman's ρ was also used to assess correlation between methylation β-values (from HM450 data) and mRNA expression *z*-scores for all genes, separating patient data by tumour stage; positive ρ values indicate that increased gene methylation can be correlated to increased gene expression, while conversely, a negative ρ values that increased gene methylation can be correlated to decreased gene expression.

### 2.3 Survival analysis

Survival analysis was performed for selected patient groups meeting specific criteria. Patients were categorized as “high” or “low” expressers of genes, using an absolute mRNA expression *z*-score cutoff of 0.75; such groups required a minimum of 10 patients before further analysis was performed. The log-rank test was used to assess whether differences in survival were statistically significant; comparisons yielding *p-*values less than 0.05 were selected as statistically significant. Patient groups with single gene high/low phenotypes were assessed, as well as groups with two gene high/low phenotypes.

### 2.4 Gene set enrichment analysis

For patient groups with phenotypes affording significant differences in survival, gene set enrichment analysis (GSEA) was performed using the GSEA software [41], [42]. The lists of patients corresponding to each patient group for comparison were manually provided to GSEA to create phenotypes for analysis. GO gene sets [43] were used in the analysis. Gene sets affording *p*-values less than 0.05 and *q*-values (false discovery rates) of less than 0.25 were selected as being significantly altered between the patient groups. The remaining gene sets were then ranked by the GSEA normalised enrichment score (NES), and up to the top 350 ranked gene sets were analysed using REVIGO [44] to further summarize the biological importance of the identified gene sets. NESs for each gene set were supplied to REVIGO as a measure of the relative significance of each gene set. The human database was used and allowed similarity of output gene sets was set to tiny (0.4). The SimRel semantic similarity measure was used [45].

## 3 Results

### 3.1 Analysis of co-expression patterns of heat shock proteins and redox regulator genes in GI carcinomas

To determine co-expression patterns of the 126 HSP and redox regulator genes, we calculated Spearman's rank correlation coefficient (ρ) for all gene combinations for the stomach adenocarcinoma, colorectal adenocarcinoma and oesophageal carcinoma datasets. Hierarchical clustering revealed numerous HSPs and redox regulators that were strongly positively and negatively correlated in all carcinoma datasets.

**Fig. 1:**
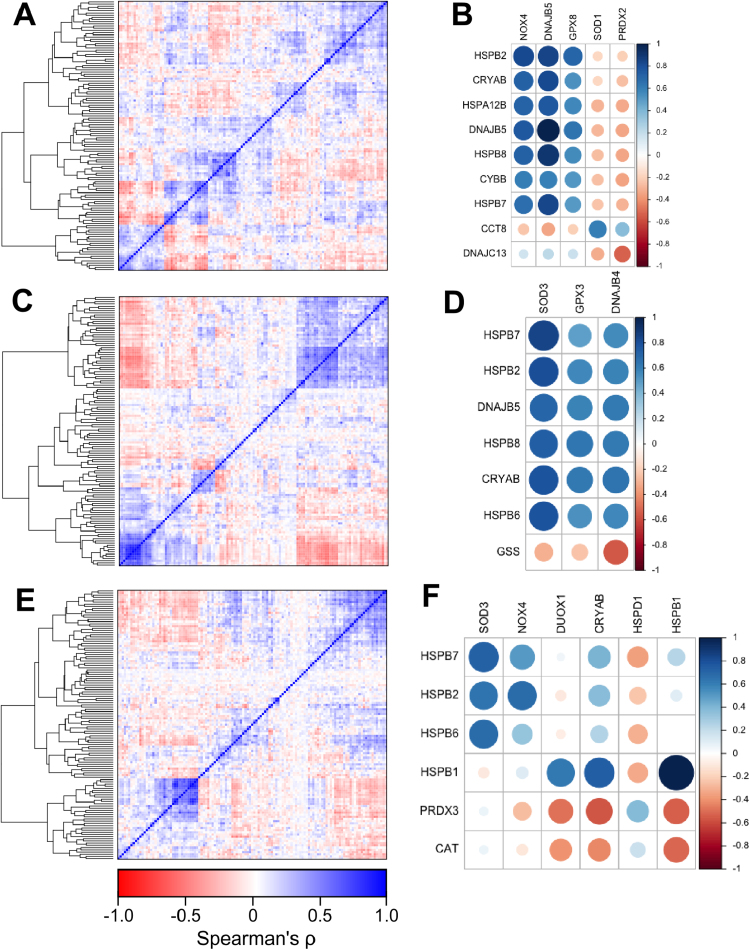
Co-expression analysis of HSPs and redox regulators in gastrointestinal tumours. Heatmaps of Spearman's rank correlation coefficient (ρ) for all HSP and redox genes (A, C,E) and selected gene combinations (B, D,F) in the following TCGA datasets: colorectal carcinoma (A, B), stomach adenocarcinoma (C, D), oesophageal carcinoma (E, F). In all panels, blue indicates negative ρ, red indicates positive ρ. In panels B, D and F, circle size indicates magnitude of ρ.

The colorectal dataset showed a strong positive correlation in gene expression between NADPH oxidase 4 (*NOX4*) and numerous members of the small HSP family, including *HSPB2, HSPB5* (*CRYAB*), *HSPB7 and HSPB8,* as well as members of the HSP40 family *DNAJB5* and *DNAJC5B. NOX4* also has a strong positive correlation with *HSPA12B*, a member of the HSP70 family (Fig. 1A & B). Other strong positive correlations in gene expression were identified between superoxide dismutase 1 (*SOD1*) with *CCT8* and glutathione peroxidase 8 (*GPX8*) with *HSPB2*. Peroxiredoxin 2 (*PRDX2*) gene expression was negatively correlated with *DNAJC13*.

In the stomach adenocarcinoma dataset, strong positive correlations in gene expression were identified between members of the small HSP family, including *HSPB2, HSPB5* (*CRYAB*)*, HSPB6, HSPB7* and *HSPB8,* as well as the HSP40 family member *DNAJB5*, with superoxide dismutase (*SOD3*) (Fig. 1C & D). Glutathione peroxidase 3 (*GPX3*) gene expression was strongly positive correlated with *HSPB8*. The only redox/HSP combination that exhibited a noteworthy negative correlation at the level of gene expression was between glutathione synthetase (*GSS*) and *DNAJB4*.

Strong positive correlations in the expression of *SOD3* with the expression of *HSPB2*, *HSPB6* and *HSPB7* were found in the oesophageal carcinoma dataset (Fig. 1E & F). Gene expression of the NADPH oxidase family members *NOX4* and *DUOX1* was positively correlated with expression of *HSPB1* and *HSPB2*. Negative correlations were found between *PRDX3* with both *HSPB5* and *HSPD1*, as well as between *CAT* and *HSPB1*.

### 3.2 Survival analysis of patient groups exhibiting differential expression of HSP and redox genes

We then set to determine if there were novel predictors of overall and disease-free survival based on combinations of HSP or redox genes, which exhibited either high or low expression. Single gene and two-gene survival analyses were performed.

**Fig. 2:**
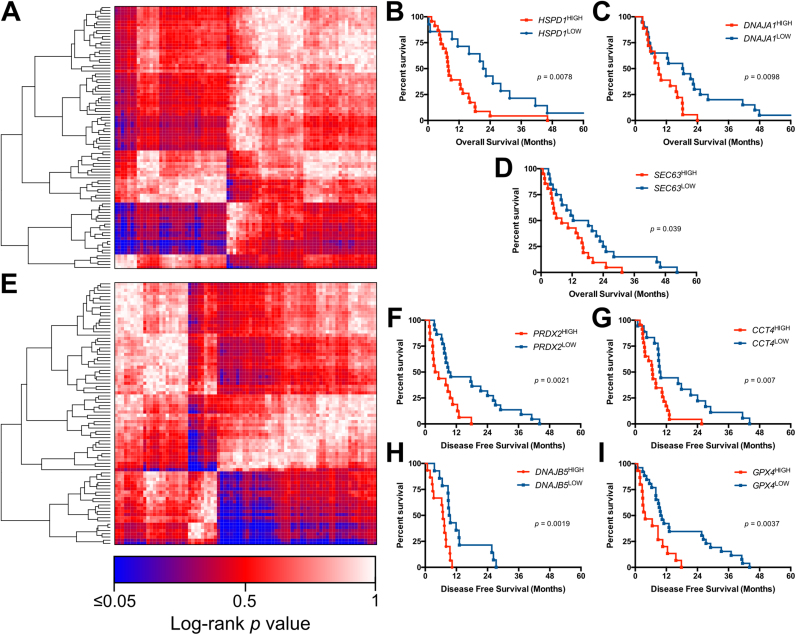
Single gene survival analysis of HSPs and redox regulators in oesophageal carcinoma. A) Heatmap of log-rank p-values for overall survival differences of HSPs and redox regulators in oesophageal carcinoma. Blue indicates significant (p < 0.05) survival differences, Red indicates non-significant (p > 0.05) survival differences. Overall survival analysis for B) *HSPD1*^high^ vs *HSPD1*^low^ C) *DNAJA1*^high^ vs *DNAJA1*^low^ D) *SEC63*^high^ vs *SEC63*^low^. E) Heatmap of log-rank p-values for disease-free survival differences of HSPs and redox regulators in oesophageal carcinoma. Overall disease-free survival analysis for F) *PRDX2*^high^ vs *PRDX2*^low^ G) *CCT4*^high^ vs *CCT4*^low^ H) *DNAJB5*^high^ vs *DNAJB5*^low^ I) *GPX4*^high^ vs *GPX4*^low^.

In the oesophageal carcinoma dataset, patients exhibiting high expression of *HSPD1*, *DNAJA1* and *SEC63* all resulted in significantly lower overall survival when compared to patients with low expression (Fig. 2A-D). High expression of *PRDX2* and *GPX4* reduced the disease-free survival significantly compared to patients with lower mRNA levels of these genes. Furthermore, patients with higher expression of *DNAJB5* and *CCT4* had significantly reduced disease-free survival rates (Fig. 2E-I). Two-gene survival analysis indicated that patients with *CCT2*^*high*^*/GSS* ^*high*^, *DNAJB12* ^*high*^*/GSS* ^*high*^ and *DNAJC8* ^*high*^*/GSS* ^*high*^ phenotypes all exhibited poorer disease-free survival, compared to patients with *CCT2*^*low*^*/GSS*^*high*^, *DNAJB12*^*low*^*/GSS*^*high*^ and *DNAJC8*^*low*^*/GSS*^*high*^ expression (Fig. 5A–C).

**Fig. 3:**
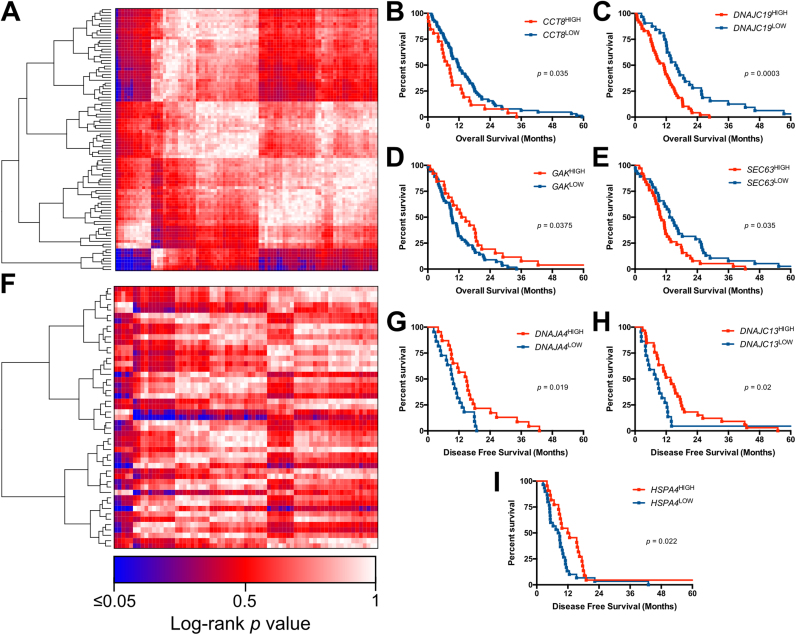
Single gene survival analysis of HSPs and redox regulators in stomach adenocarcinoma. A) Heatmap of log-rank p-values for overall survival differences of HSPs and redox regulators in stomach adenocarcinoma. Blue indicates significant (p < 0.05) survival differences, Red indicates non-significant (p > 0.05) survival differences. Overall survival analysis for B) *CCT8*^high^ vs *CCT8*^low^ C) *DNAJC19*^high^ vs *DNAJAC19*^low^ D) *GAK*^high^ vs *GAK*^low^ E) *SEC63*^high^ vs *SEC63*^low^. F) Heatmap of log-rank p-values for disease-free survival differences of HSPs and redox regulators in stomach adenocarcinoma. Disease-free survival analysis for G) *DNAJA4*^high^ vs *DNAJA4*^low^ H) *DNAJC13*^high^ vs *DNAJC13*^low^ I) *HSPA4*^high^ vs *HSPA4*^low^

The stomach adenocarcinoma dataset revealed that overall survival was significantly altered as a result of differential expression of four HSPs - *CCT8*, *DNAJC19*, *GAK* and *SEC63* (Fig. 3B-E). Low expression of *CCT8*, *DNAJC19* and *SEC63* significantly increased overall survival, whereas low expression of *GAK* significantly decreased overall survival (Fig. 3D). Significantly poorer disease-free survival rates were found in patients with high *DNAJA4*, *DNAJC13* and *HSPA4* expression compared to patients with low expression (Fig. 3G-I). Two-gene survival analysis revealed significantly poorer survival in patients with *CCT5*^*high*^*/GPX1*^*low*^ expression compared to *CCT5*^*low*^*/GPX1*^*low*^ expression. Conversely, patients with *DNAJC7*^*high*^*/GPX1*^*low*^ expression had a significantly better disease free survival rate than those with *DNAJC7*^*low*^*/GPX1*^*low*^ expression. High expression of both *CCT4* and *CCT7* resulted in significantly poorer survival than those with low expression of *CCT4* and *CCT7* (Fig. 5D–F).

**Fig. 4:**
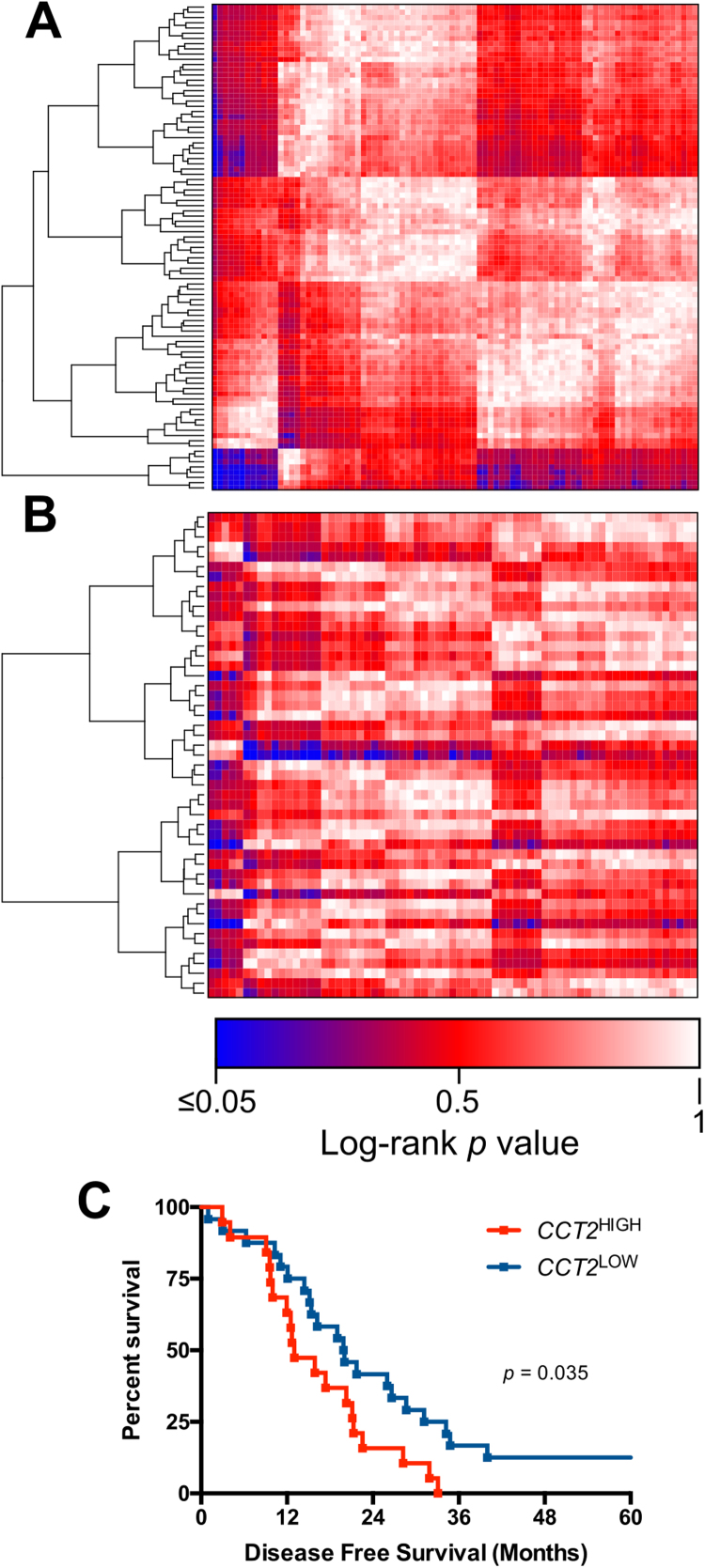
Single gene survival analysis of HSPs and redox regulators in colorectal carcinoma. A) Heatmap of log-rank p-values for overall survival differences of HSPs and redox regulators in colorectal carcinoma. B) Heatmap of log-rank p-values for disease-free survival differences of HSPs and redox regulators in colorectal carcinoma. Blue indicates significant (p < 0.05) survival differences, Red indicates non-significant (p > 0.05) survival differences. C) Disease-free survival analysis for *CCT2*^high^ vs *CCT2*^low^.

**Fig. 5:**
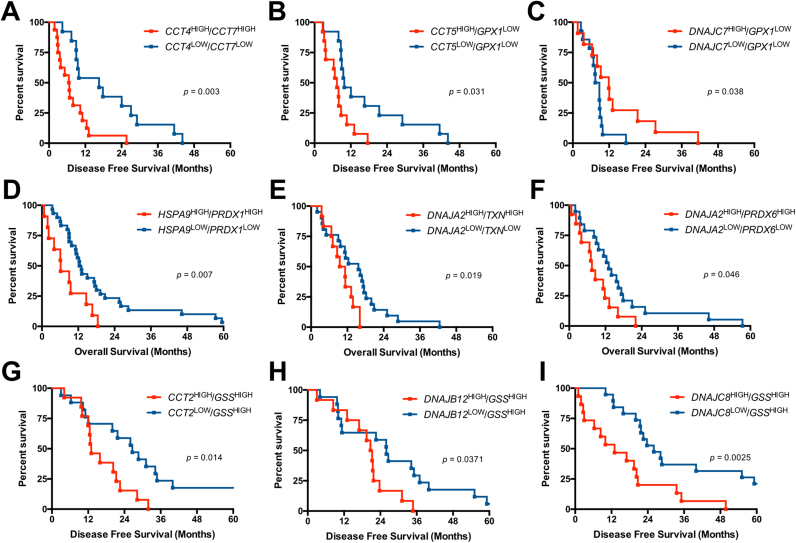
Combinatorial gene survival analysis of HSPs and redox regulators in gastrointestinal cancers. Disease-free survival analysis for A) *CCT4/CCT7*^high^ vs *CCT4/CCT7*^low^, B) *CCT5/GPX1*^high^ vs *CCT5/GPX1*^low^ C) *DNAJC7/GPX1*^high^ vs *DNAJC7/GPX1*^low^ in oesophageal carcinoma dataset. Overall survival analysis for D) *HSPA9/PRDX1*^high^ vs *HSPA9/PRDX1*^low^ E) *DNAJA2/TXN*^high^ vs *DNAJA2/TXN*^low^ and F) *DNAJA2/PRDX6*^high^ vs *DNAJA2/PRDX6*^low^ in stomach adenocarcinoma dataset. Disease-free survival analysis for G) *CCT2*^high^/*GSS*^high^ vs *CCT2*^low^/*GSS*^low^ H) *DNAJB12*^high^/*GSS*^high^ vs *DNAJB12*^low^/*GSS*^low^ I) *DNAJC8*^high^/*GSS*^high^ vs *DNAJC8*^low^/*GSS*^low^ in colorectal carcinoma dataset.

The colorectal adenocarcinoma dataset revealed that the increased expression of *CCT2* resulted in a significantly decreased disease-free survival (Fig. 4C). When two-gene survival analysis was performed combinations of HSP and redox regulators, patients with *HSPA9*^*high*^*/PRDX1*^*high*^*, DNAJA2*^*high*^*/TXN*^*high*^ and *DNAJA2*^*high*^*/PRDX6*^*high*^ genotypes all had significantly decreased overall survival rates when compared to the same combinations with low expression (Fig. 5G–I).

### 3.3 Correlations between gene methylation and expression at different pathological stages

**Fig. 6:**
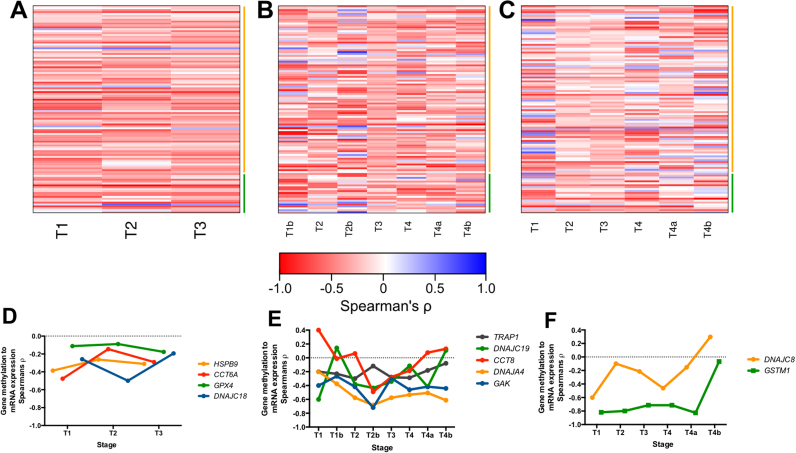
Correlation of methylation and gene expression of HSPs and redox regulators in gastrointestinal cancers. Heatmap of Spearman's rank correlation coefficient (ρ) for TCGA datasets of A) oesophageal carcinoma B) stomach adenocarcinoma and C) colorectal carcinoma for various tumour stages (T1-T4b). Blue indicates negative Spearman's coefficient, Red indicates positive Spearman's coefficient. Green line indicates redox regulator genes, yellow line indicates HSP genes. D) Line graph plotting gene methylation to mRNA expression through tumour stages T1-T3 of *HSPB9, CCT6A, GPX4* and *DNAJC18* for oesophageal carcinoma dataset. E) Line graph plotting gene methylation to mRNA expression through tumour stages T1-T4b of *TRAP1, DNAJC19, CCT8, DNAJA4* and *GAK* for stomach adenocarcinoma dataset. F) Line graph plotting gene methylation to mRNA expression through tumour stages T1-T4b of *DNAJC8*, and *GSTM1* for colorectal carcinoma dataset.

**Fig. S4:**
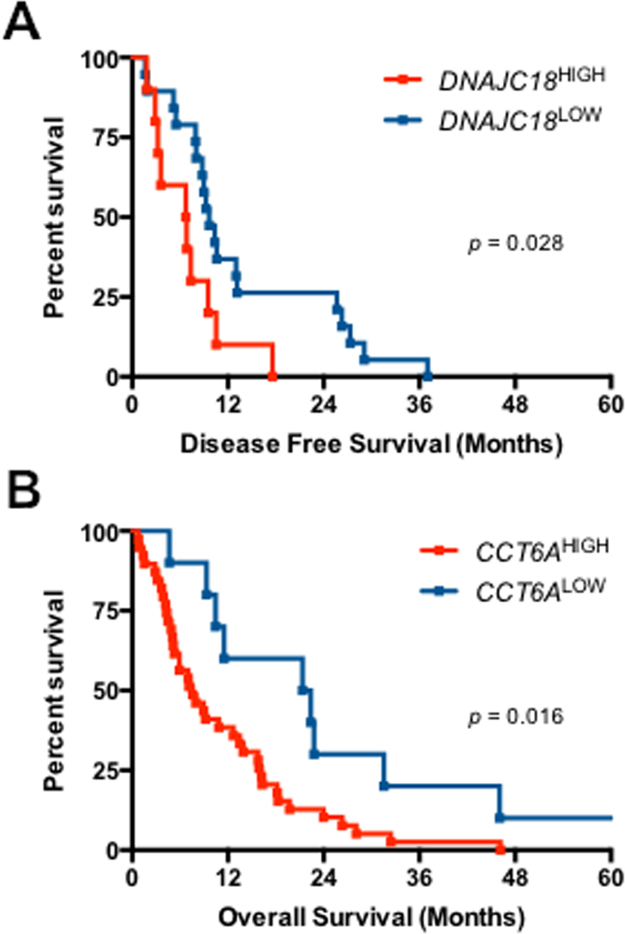
A) Disease-free survival analysis for *DNAJC18*^high^ vs *DNAJC18*^low^ patients in the esophageal carcinoma dataset. B) Overall survival analysis for *CCT6A*^high^ vs *CCT6A*^low^ patients in the oesophageal carcinoma dataset.

We set to determine Spearman correlation coefficients between gene methylation and expression for 126 genes in our HSP and redox regulator set, and to draw connection between this, pathological stage and survival (Fig. 6A-C). The majority of genes in oesophageal carcinoma unexpectedly exhibited a negative correlation between methylation and mRNA expression at stages T1, T2 and T3 (Fig. 6D). *HSPB9* showed a negative correlation across these stages (Fig. 6D). *GPX4* demonstrated a weak negative correlation that remained mainly unchanged across different pathological stages. *CCT6A* exhibited the strongest negative correlation at T1, which decreased with tumour stage progression. *DNAJC18* showed a strong negative correlation at T2, which progressed to a weak negative correlation in stage T3. The loss of a strong negative correlation during the progression of tumours suggests that there may be decreased methylation and increased mRNA expression during the more progressed stages. This is consistent with survival data for *DNAJC18* (Fig. S4A) and *CCT6A* (Fig. S4B), both of which, when highly expressed, result in significantly poorer overall patient survival compared to patients with low expression. Conversely, consistently low negative correlations between stages in *HSPB9* suggest no loss of methylation and low mRNA expression, consistent with low levels of *HSPB9* reflecting significantly poorer patient survival.

When the analysis was performed on the stomach adenocarcinoma data, a strong negative correlation between gene expression and methylation was observed for *DNAJA4*. This correlation was stronger in the later stages (T2, T2b, T3, T4, T4a and T4b) than in T1 (Fig. 6E). The survival analysis revealed that patients with *DNAJA4*^*low*^ had a significantly poorer disease free survival than those with the *DNAJA4*^*high*^ phenotype, suggesting that increased gene methylation of *DNAJA4* may predict poorer survival. A similar trend of a strong negative correlation was seen in *GAK* in all stages (T1-T4b). Again, patients with a *GAK*^*low*^ phenotype had a significantly poorer overall survival than those with a *GAK*^*high*^ phenotype, suggesting that the methylation status, corresponding to overall gene expression, plays an important role in overall patient survival.

In the colorectal dataset, two genes were identified where methylation may play an important role in gene expression at different tumour stages. *GSTM1* exhibited a very strong negative correlation between methylation and gene expression in stages T1 through to T4a, however, this correlation was nearly completely lost in stage T4b (Fig. 6F). Methylation of *DNAJC8* was strong negatively correlated in stage T1, suggesting gene silencing through methylation, although in T4a and T4b, this correlation was reversed, suggesting that methylation was correlated with an increase in gene expression.

### 3.4 Gene set enrichment analysis in patient groups associated with significantly altered survival

**Fig. 7:**
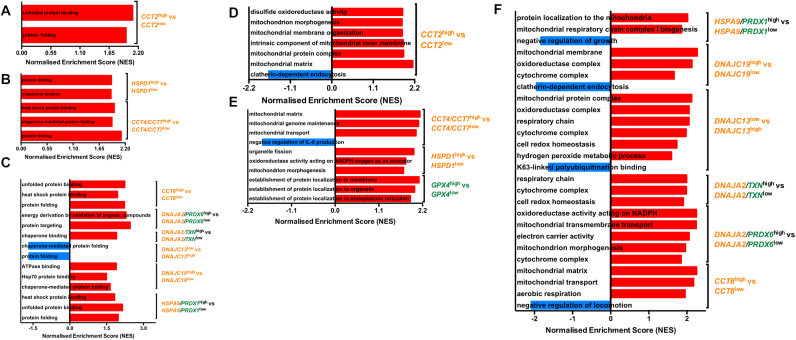
Gene set enrichment analysis (GSEA) of HSPs and redox regulators in gastrointestinal cancers. Bar graphs of normalised enrichment scores (NES) from GSEA of various HSP^high^ vs HSP^low^ phenotypes in A) colorectal carcinoma B) oesophageal adenocarcinoma and C) stomach adenocarcinoma for validation of HSP related processes. Bar graphs of normalised enrichment scores (NES) from GSEA of various redox regulators^high/low^/HSP^high/low^ vs redox regulators^high/low^/HSP^low/high^ phenotypes in D) colorectal carcinoma E) oesophageal carcinoma and F) stomach adenocarcinoma. Heat shock protein genes indicated by yellow writing, redox regulators indicated by green writing.

To determine whether the genotypes associated with differing disease-free or overall survival outcomes were also broadly associated with other biological processes and molecular functions, we performed gene set enrichment analysis (GSEA). The Gene Ontology (GO) gene sets were used for this purpose. Across the three gastrointestinal tumour types, grouped individuals with high expression of HSP family members were, unsurprisingly, enriched in gene sets covering processes involved in canonical HSP function, for example, protein folding, chaperone-mediated protein folding and heat shock protein binding (Fig. 7A–C).

The colorectal adenocarcinoma dataset, which had only one statistically significant survival phenotype (*CCT2*^high^ vs *CCT2*^low^), was primarily enriched in GO gene sets involved in mitochondrial transport and organisation. (Fig. 7D). *CCT2*^low^ individuals were enriched for gene sets involved in clathrin-mediated endocytosis compared to *CCT2*^high^ individuals.

GSEA on the oesophageal carcinoma phenotypes associated with differential survival outcomes were enriched in GO terms associated with mitochondrial matrix, transport and genome maintenance (*CCT4/CCT7*^high^ vs *CCT4/CCT7*^low^), mitochondrial morphogenesis and oxidoreductase activity (*HSPD1*^high^ vs *HSPD1*^low^) and protein localisation to the membrane, organelles and ER (*GPX4*^high^ vs *GPX4*^low^). The *CCT4/CCT7*^low^ vs *CCT4/CCT7*^high^ phenotypes were enriched for gene sets involved in the negative regulation of IL-6 production (Fig. 7E).

In the stomach adenocarcinoma dataset, GO gene sets for mitochondrial membrane transport and structure, as well as mitochondrial respiration components, were significantly enriched. Gene sets involved in hydrogen peroxide metabolic processes and cell redox homeostasis were enriched in patients with a *DNAJC13*^*l*ow^ vs *DNAJC13*^*high*^ phenotypes. *HSPA9*^high^/*PRDX*1^high^ individuals were significantly enriched in genes mediating mitochondrial respiratory chain complex I biogenesis and protein localisation to the mitochondria, while conversely, *HSPA9*^low^/*PRDX*1^low^ individuals were significantly enriched for gene sets involved in the negative regulation of growth. Gene sets for mitochondrial matrix, transport and aerobic respiration were all enriched in the *CCT8*^high^ individuals, while genes mediating negative regulation for locomotion were enriched in grouped individuals demonstrating a *CCT8*^low^ phenotype (Fig. 7F).

## 4 Discussion

We analysed publicly available TCGA datasets from three gastrointestinal tumours utilising a list of 96 HSP family genes and 30 genes that have been implicated in cellular redox functions. We analysed gene correlations, correlations between methylation status in tumour stages with gene expression, and overall and disease free survival in patients. We then selected the genotypes that demonstrated significant differences in patient survival and performed gene set enrichment analysis to reveal up or downregulated gene sets associated with these phenotypes. The colorectal and oesophageal dataset revealed positive gene correlations between *NOX4* and a variety of HSP family members. *NOX4* has been previously shown to mediate the expression of *HSP27* mediated by TGFβ [46]. This is in line with previous reports that have demonstrated co-localisation of NOX4 with HSP27 and HSP70 in vascular smooth muscle cells [47] and myoblasts [48] respectively. In all datasets, members of the superoxide dismutase family, which are responsible for the production of H_2_O_2_ through the dismutation of O_2_^-^, have a positive correlation with many HSPs. This is consistent with previous observations that have demonstrated the induction of a variety of HSPs, including HSP30, HSP90 and HSP70, through H_2_O_2_ [49], [50], [51]. HSP70 itself directly interacts with SOD2 and may chaperone the enzyme to the mitochondria, although the exact mechanism remains unclear [52]. Interestingly, enzymes involved in ROS detoxification, such as catalase and peroxiredoxins, exhibited a negative correlation with HSPs. This aligns with the observation that *HSP* gene expression is induced by H_2_O_2_. Higher levels of intracellular hydrogen peroxide, as a result of decreased catalase or peroxidredoxin expression, may facilitate cellular redox conditions permissible for the induction of high levels of HSP family members. Strong positive correlations were shown between various members of the small HSP family and redox-related genes. It has previously been shown that HSP27 plays roles in the pathological state of cancer cells in response to oxidative stress through modulation of the ROS-glutathione pathway [53]. HSP27 has been shown to act as a protectant against the oxidation of proteins [53]. Overexpression of HSP27 has also been shown to increase extracellular O_2_^-^ production to produce a pro-oxidant state beneficial for cell survival [53], [54]. Furthermore, phosphorylation-mediated downregulation of HSP27 was shown to sensitize cancer cells to TRAIL-mediated apoptosis upon exposure to a small molecule [55] that signals through extracellular H_2_O_2_ production [56]. These observations align with results demonstrated in all these gastrointestinal cancer datasets.

Gene methylation is widely known to influence gene expression [57]. We observed a strong negative correlation between *DNAJA4* gene methylation and expression in stomach adenocarcinoma, which suggested that methylation of *DNAJA4* may suppress gene expression. This is consistent with reports that *DNAJA4* expression is strongly influenced by its methylation status [58]. Our data also revealed that patients with low *DNAJA4* expression had poorer survival outcomes. Together, this data suggests that gene methylation of *DNAJA4* may suppress its expression, leading to poorer patient outcomes. *DNAJA4* has been shown to promote metastasis and angiogenesis in melanoma [59], but may act as a tumour suppressor in stomach cancer. *DNAJA4* and its methylation status may be a new biomarker for stomach adenocarcinoma, although this may not be applicable to other carcinomas. Gene expression of *GAK* (cyclin G-associated kinase) was also found to be negatively correlated in stomach cancer, and patients with a *GAK*^*low*^ phenotype had poorer overall survival. While *GAK* has been shown to promote oncogenesis in some cancers [60], in stomach cancer it appears that higher expression of *GAK* may be beneficial*. GAK* has been shown to be essential for clathrin-mediated endocytosis [61], which, if impaired, can lead to prolonged cell signalling and higher rates of proliferation [62]. In the colorectal dataset, *GSTM1* demonstrated a high negative correlation in all stages prior to stage T4a, whereas in stage T4b this was completely lost, suggesting that low levels of GSTM1 are associated with the onset and progression of colorectal cancer. This has been confirmed in different populations, where the *GSTM1* null variant was correlated with increased colorectal cancer risk [63], [64]. The mechanisms of *GSTM1* gene methylation may provide interesting insights into the onset of colorectal cancer.

Our gene set enrichment data implicated many biological processes that are crucial for tumorigenesis in these cancer types surveyed, many being involved in mitochondrial functions, an organelle crucial in the progression of cancer [65]. In the colorectal dataset, the *CCT2*^high^ phenotype, which displayed poorer survival, was enriched in gene sets involved in mitochondrial organisation and membrane processes. While CCT2 overexpression has previously been shown to adversely affect colorectal patient survival [66], its role in mitochondrial membrane organisation is less clearly understood. A recent study in colorectal cancer identified *CCT2* as a synthetic lethal partner to mutant *KRAS*, which was found to be dependent on mitochondrial translation for viability [67]. Similarly, in oesophageal cancer, patients with *HSPD1*^*high*^ and *CCT4*^*high*^*/CCT7*^high^ phenotypes displayed enrichment for gene sets involved in mitochondrial processes, such as organelle fission, transport and genome maintenance, which were associated with poorer survival. HSPD1 was identified as an enriched gene in the ESCC4 subtype of oesophageal carcinoma, which also has the poorest survival outcomes [68]. HSPD1 has been previously shown to be critical for mitochondrial protein folding [69]. It also functions as a protectant against pharmacologically-induced oxidative stress [70]; therefore, combination therapy that initiates oxidative stress-induced apoptosis, such as myrtucommulone while simultaneously inhibiting HSP60 may result in better outcomes [71]. Glutathione peroxidase family member GPX4 protects against lipid peroxidation and ferroptosis, and is predominantly expressed in the mitochondria of the testis [72], [73]. Overexpression of GPX4 in oesophageal cancer patients correlates with the upregulation of gene sets involved in protein localisation to the ER and membrane, a process that, if dysregulated, can contribute to carcinogenesis [74]. *GPX4* overexpression may represent a novel biomarker and a potential therapeutic target for the treatment of oesophageal cancer.

The stomach adenocarcinoma dataset revealed many combinations of HSPs and redox regulators that influenced patient survival. These were also associated with many biological processes involved in mitochondrial transport, metabolism and cellular redox homeostasis. The overexpression of *DNAJC19*, which encodes for the protein TIM14, has yet to be shown to influence oncogenesis or patient survival in stomach cancer. It functions as a translocase on the inner mitochondrial membrane [75], and GSEA on *DNAJC19*^high^ patients revealed enrichment of gene sets for mitochondrial membrane and oxidoreductase activity. Conversely, *DNAJC13* overexpression may benefit patient survival outcomes. The primary role of *DNAJC13* is in early endosome transportation, [76] and in *ERBB2* positive breast cancers has been implicated in the trafficking of epidermal growth factor receptor (EGFR) to the plasma membrane [77]. In this context, *DNAJC13* is seen as a potential therapeutic target, whereas the situation is reversed in stomach cancer; the enriched gene sets for the *DNAJC13*^low^ phenotype primarily included cell redox homeostasis and those involved in mitochondrial complexes and metabolism. Therefore, *DNAJC13* depletion may facilitate mitochondrial complex function and pro-oxidant state necessary to promote tumorigenesis. *CCT8*^high^ individuals were found to have a significantly poorer overall survival, and interestingly, the GSEA revealed that *CCT8*^low^ individuals were enriched for gene sets involved in the negative regulation of locomotion. This is consistent with the reduction in cell migration and invasion observed in glioma cells following siRNA-mediated knockdown of *CCT8* [78].

This study has uncovered a variety of novel HSP and redox genes that may influence patient outcomes and survival in gastrointestinal cancers. This high-throughput method of data extraction and analysis will become more valuable as more patient data becomes available. This study also sets a foundation for the analysis and development of multi-gene signatures that can predict patient survival, and exponentially expand our list of biomarkers for the onset and progression of a variety of cancers.

## Appendix A Supplementary material

Data S1. Correlation co-efficients for paired gene co-expression in all colorectal adenocarcinoma (COAD), oesophageal carcinoma (ESCA) and stomach adenocarcinoma (STAD) sets, ordered as depicted in Figs. 1A, 1C, and 1E.

Data S2. Correlation co-efficients between methylation and gene expression for patients at progressing stages of colorectal adenocarcinoma (COAD), oesophageal carcinoma (ESCA) and stomach adenocarcinoma sets (STAD), ordered as depicted in Figs. 5A, 5B and 5C.

Data S3. p-values for log-rank tests on overall (os) and disease-free survival (dfs) in high- vs low-expressing colorectal adenomcarcinoma (crc), oesophageal carcinoma (eso) and stomach adenocarcinoma sets (stom), ordered as depicted in Figs. 2A, 2E, 3A, 3F, 4A and 4B.
